# Introduction to the Study and Practice of Midwifery

**Published:** 1845-07

**Authors:** 


ART. VIII.—*Introduction to the Study and Practice of Midwifery.* By W. CAMPBELL, Late Surgeon R.N., and by ALEXANDER D. CAMPBELL, B.A. Trin. Coll. Dub., &c. 2d Edit.—*Edinb.* and *Lond.*, 1843. 8vo, pp. 800.

WE have to apologize to the authors of this book, for having so long neglected to notice it. The omission to do so at the time of its publication, arose from an accident; and now, when we are prepared to rectify our oversight, our arrangements preclude us from giving more than a mere notice of its contents and general character. In it are embraced all the various subjects belonging to the practice of midwifery: the anatomy and physiology of the female organs of generation in the unimpregnated and gravid states; the medico-legal questions connected with them; the pathology and treatment of the diseases incidental to women and children. Without containing any very novel views or important additions to practice or science, it may fairly be characterized as constituting a safe guide, and a good epitome of practical information, which may be perused with advantage by those who are commencing either the study or practice of midwifery.

